# Preparing to Respond to the Next Pandemic: Impact of Key WHO IPC COVID‐19 Response Products

**DOI:** 10.1002/puh2.70133

**Published:** 2025-09-30

**Authors:** Femi Nzegwu, Hannah Hamilton Hurwitz, Victoria Willet, Elizabeth Clery, Maryirene Ibeto, Joanna Wild, April Baller

**Affiliations:** ^1^ Department of Infectious Disease Epidemiology & Dynamics London School of Hygiene & Tropical Medicine London UK; ^2^ Health Emergencies Programme World Health Organization Geneva Switzerland; ^3^ Freelance Social Scientist London UK; ^4^ Department of Education London School of Hygiene & Tropical Medicine London UK; ^5^ OL4ALL Oxford UK

**Keywords:** COVID‐19 pandemic response, health emergencies preparedness, infection prevention and control, lessons learned, mixed methods study, outcomes/impact evaluation, pandemic preparedness

## Abstract

An outcomes/impact evaluation was undertaken (July 2023–April 2024) to evaluate the work of the World Health Organization (WHO) infection prevention and control (IPC) coronavirus disease (COVID‐19) response team during the COVID‐19 pandemic and to identify how to better prepare for the next pandemic. The evaluation used mixed methods, focusing on three pillars of the team's work: (1) interim guidance and guidelines; (2) OpenWHO IPC courses; and (3) risk communication and community engagement (RCCE) interventions. A literature review, interviews with WHO headquarters and regional staff, case studies, and multicountry surveys were undertaken. Of the national representatives surveyed, 97% reported that the guidelines impacted their work; overall 76% of Member States used RCCE interventions and products; and 99% of course participants used what they learned professionally or personally. Member States identified gaps relating to national contextualization, availability of feedback and monitoring mechanisms, and knowledge sharing. There is strong evidence of the impact, effectiveness, and use of this team's work in developing IPC COVID‐19 guidelines and through courses developed and shared via OpenWHO; however, there is weaker evidence on the impact of RCCE products/interventions as a standalone activity. To be better prepared for the next pandemic, Member States and the WHO need to collectively identify how best to sustain the IPC gains and address the gaps.

## Graphical abstract

GRAPHICAL ABSTRACT

An evaluation exploring the impact of the WHO IPC COVID‐19 response team's work. The mixed methods evaluation reviewed: WHO guidelines, OpenWHO courses, and RCCE products. Member States identified gaps in contextualization, availability of feedback mechanisms, and knowledge sharing. To prepare for a future pandemic, gains must be sustained and gaps addressed.

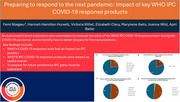

## 1 Introduction

The World Health Organization (WHO) health emergencies programme (WHE) infection prevention and control (IPC) and water, sanitation, and hygiene (WASH) team's mandate is “to strengthen capacities of countries and their health systems for improved emergency preparedness, operational readiness, and response through evidence‐based IPC and WASH measures in healthcare and community settings while mitigating healthcare‐associated infections.^[^ ^1^ ^]^” Between July 2023 and April 2024, an outcomes/impact evaluation and lessons learned study was undertaken by the UK Public Health Rapid Support team with the support of the WHE IPC team to evaluate the work of the WHO IPC coronavirus disease 2019 (COVID‐19) response team (hereafter referred to as “the team”) during the COVID‐19 pandemic (January 2020–May 2023) with the objective of identifying areas of improvement and therefore better prepare for the next pandemic.

The primary purpose of the evaluation was to generate in‐depth learning about the impact, effectiveness, efficiency, and sustainability of the IPC workstreams being reviewed; and specifically, to understand the degree to which the team's activities impacted Member States. The evaluation focused on three core IPC work pillars: IPC guidance/guidelines, risk communication & community engagement (RCCE) interventions, and IPC courses on the OpenWHO^[^ ^1^ ^]^, all developed in support of Member States.^[^ ^2^ ^]^

## 2 Methods

This was a mixed methods study examining the impacts of three core IPC pillars of work from headquarters, regional, and country‐level perspectives. The overall approach was underpinned by three evaluation principles to ensure a fully participatory approach. Accordingly, the evaluation was: (1) utilization focused^[^ ^3^ ^]^ endeavoring to promote a strong sense of engagement and ownership of both the process and findings among participants—providing actionable findings, insights, and learning to inform the team's collective work in the future; (2) structured around an evaluation framework that considered four OECD DAC^[^ ^4^ ^]^ evaluation criteria (efficiency, effectiveness, impact, and sustainability); seven study questions, which together provided the focus for all data collection and analysis; and (3) delivered as a collaborative learning experience for country‐level participants who co‐designed and co‐implemented the in‐country work. Finally, key lessons learned were generated from the overall review of the team's work.

The three core IPC pillars were explored through a rapid review of internal and external documents, in‐depth interviews, case studies, focus group discussions, surveys, and OpenWHO training data. A rapid review of key academic literature, grey literature, and internal reports was performed to situate the study more broadly in the wider work of the IPC sector. Forty‐seven documents and 14 peer‐reviewed publications, in which the team was involved, were reviewed. All survey and interview data were anonymized and kept confidential. Participation was voluntary, with no personally identifiable information collected. Interviewees gave informed consent, and data storage procedures on secure servers were explained both in writing and verbally. The aim of the internal documents review was threefold: (1) to understand the nature and extent of work completed by the team between January 2020 and May 2023, (2) to inform the design of the data collection instruments, and (3) to collect existing evidence of outcomes and impact of the work carried out by the team to achieve its mandate. The external literature review aimed to identify what was known about the impact, effectiveness, and efficiency of the work undertaken by the WHO COVID‐19 response team, other IPC teams, and organizations, in relation to COVID‐19 between 2020 and 2023.

In‐depth interviews and focus group discussions were undertaken with members of the WHO IPC COVID‐19 team, the WHO RCCE team, and OpenWHO teams, who worked collaboratively on the three pillars of IPC COVID‐19 response work. Further interviews were undertaken with individuals who worked on IPC, primarily as focal points, in the six WHO regions. In total, 20 interviews were conducted across the WHO's headquarters and regional offices to provide insight into the work undertaken on the core pillars. Interviews with regional staff aimed to understand how these products were received at the regional level and how they were disseminated (and received) at the national level. Interviews lasted between 60 and 90 minutes on average, were recorded and transcribed, and were subjected to inductive analysis around five themes (including learning) in line with the evaluation questions and framework.

Case studies were undertaken in two countries—Namibia and Nepal—based upon interest and the recommendation of WHO regional offices. An outcome harvesting approach^[^ ^5^ ^]^ was used to identify whether and how impacts were created by the team's work in these two countries. Evidence was collected on outcomes that had been achieved, and, working backward through an inductive approach, the evaluation team documented whether and how the IPC team's work had contributed to the identified outcomes.

Two separate surveys were administered between February and April 2024. One was directed at WHO regional staff and a second at the national Ministry of Health (MoH) staff and others who were likely to have used the materials produced by the team. The surveys were designed to gather quantitative data on their attitudes to and experiences of these materials and were shared online via SurveyMonkey. To compare the attitudes and experiences of regional and national representatives, questions were constructed to be as equivalent as possible, where appropriate. At the start of the survey, respondents were asked which of the WHO COVID‐19 IPC pillars of work (guidance/guidelines, OpenWHO training, and/or RCCE products) they used as part of their work; they were subsequently directed to only complete the sections relevant to the selected items. The surveys contained a mixture of quantitative and qualitative questions, with the latter generally exploring the reasons for the former.

Limitations of this study include: (1) Findings are based on a moderate number of interviews; (2) interviews are indicative and directional rather than definitive; (3) the duration of the period under study (January 2020–May 2023); (4) the time that elapsed between that period and the study may limit participants’ capacity to recall events accurately; and (5) survey results are invariably somewhat skewed due to low numbers of respondents. Although these may limit the degree of generalization, these findings are substantiated through data triangulation. We also acknowledge the contributory role of other international and national technical agencies to the progress and impacts observed in this study.

## 3 Results

### 3.1 SECTION 1: Impact of IPC COVID‐19 Response Products

To differing degrees, three sets of products developed by the team during the COVID‐19 pandemic were regarded as having substantial and sustainable impacts on IPC practices by those at the global, national, and local levels. The discussion begins with a brief review of these impacts, followed by an exploration of ongoing challenges that should be addressed to enhance the effectiveness of future pandemic responses.

#### 3.1.1 Guidelines

**FIGURE 1:**
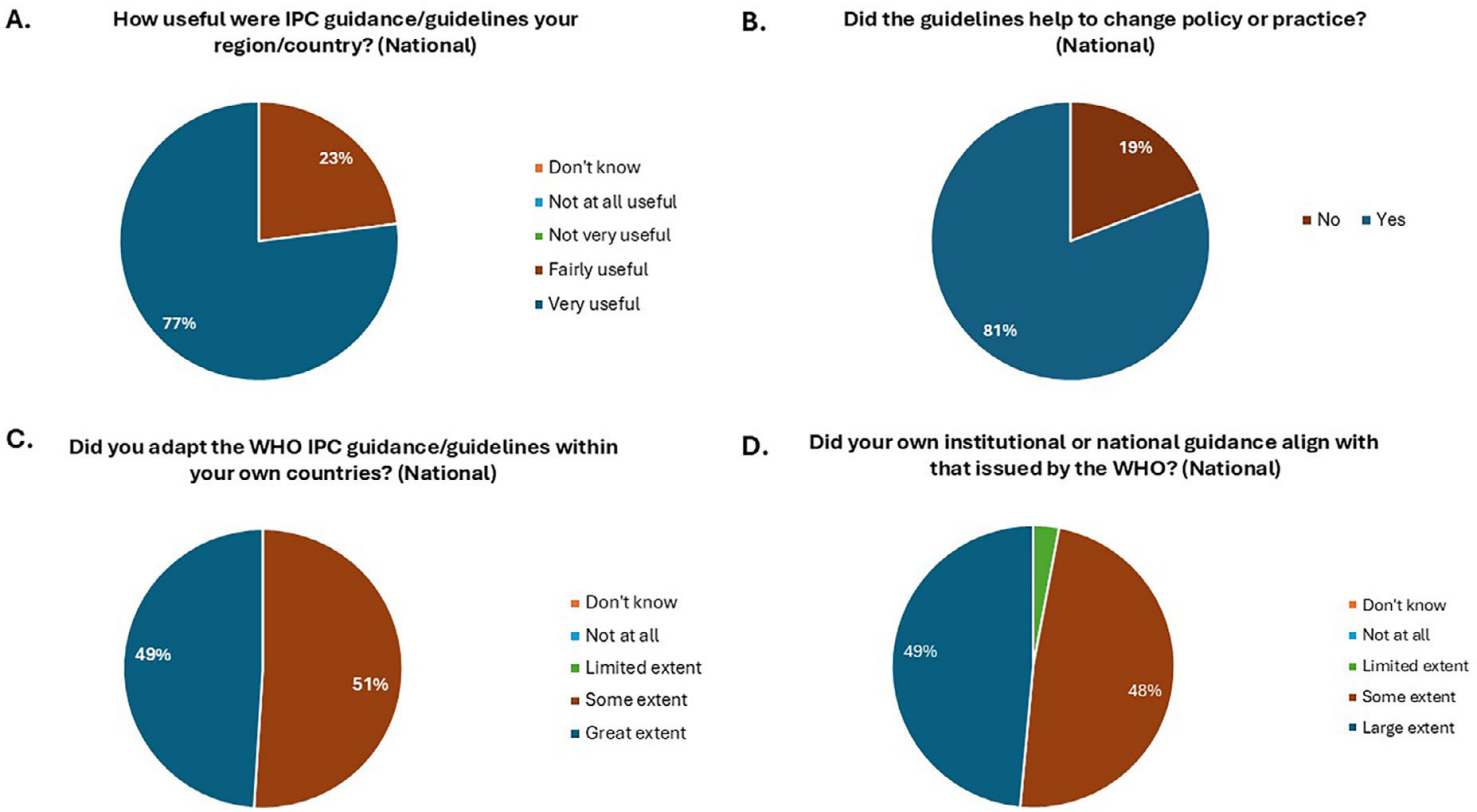
Impacts of WHO IPC COVID‐19 guidance/guidelines at the national level. In summary, the national IPC representatives found WHO guidelines to be useful, that the WHO guidelines contributed to policy change and were adapted and adopted nationally. Furthermore, the national and institutional guidelines generally aligned with WHO guidance. IPC, infection prevention and control; WHO, World Health Organization. Figure 1A depicts the perceived usefullness of the guidelines; Figure 1B reflects if the participants felt the guidelines helped change national policy/practice; Figure 1C reflects if participants felt the guidelines were adpated for their own contexts; and, Figure 1D reflects the perception of national guidelines' alliance with WHO guidelines.

Among the 35 participants working at the national level, 77% (27/35) reported that the 13 sets of guidelines issued by the WHO during the COVID‐19 pandemic had been “very useful” for their country, whereas the remainder (23%; 8/35) assessed them as having been “fairly useful” (Figure 1). The balance of opinion among those working at the regional level was very similar. Almost all national representatives felt the guidelines had helped to change policy or practice in either a health institution or at a national level within their country. All six regional participants indicated that policy/practice change had happened within their regions. When elaborating on these changes, national representatives discussed awareness being raised, leading to changes in behavior and, in their view, supporting reductions in mortality and morbidity in health facilities. Overall, there is evidence of positive change in governance structures; national policy and strategy development being enabled by the guidance/guidelines; national guidelines being aligned with guidance/guidelines, often alongside guidance from other centers of excellence; health and care worker capacities being significantly enhanced in the area of IPC; and IPC cultures within health facilities being strengthened as awareness of the guidance/guidelines occurred and they became a reference point for discussions in IPC committees and working groups nationally.

#### 3.1.2 Training^[^ ^6^ ^]^

Four online courses were developed by the team during the COVID‐19 pandemic:

*How to put on and remove personal protective equipment (PPE); Guidance on mask use in the context of COVID‐19; Prevention, identification, and management of infections in health workers in the context of COVID‐19; Infection Prevention and control for COVID‐19 Virus*. A fifth course, *Long term care facilities in the context of COVID‐19*, was developed by the WHO Western Pacific region. Courses were hosted on the OpenWHO platform, on a designated IPC channel, where they were publicly available. Courses were designed for low bandwidth access, primarily consisting of PowerPoint slides, voiceovers, video demonstrations, and quizzes. They were asynchronous in nature, reflecting the need of individuals in a range of different time zones to be able to access them quickly.

These courses were widely accessed, with the PPE course attracting close to half a million participants and the IPC for COVID‐19 virus course nearly a million participants. However, completion rates varied widely, from 55% (a total of 483,818 completers) for the IPC for COVID‐19 virus course to 85% (369,907) for the PPE course. Nor was the geographic spread of participation uniform: participants were mainly English and Spanish speaking, despite OpenWHO translating the courses into several languages (translations varied; some were translated into the six official UN languages^[^ ^3^, ^6^, ^7^ ^]^, but the core IPC course was available in 24 distinct languages).

Although large numbers completed the course, this does not necessarily reflect impact.

The training surveys reveal some impact of the WHO courses on learners. Among survey respondents, assessments of the four courses were highly positive (Table 1). Around two‐thirds of English‐speaking participants felt they had used what they had learned from each of the courses to “a great extent” as part of their work, studies, or private life during the COVID‐19 pandemic, compared with over three‐fourths of Spanish‐speaking participants (Table 1). Data are not routinely collected beyond the point of course completion, making it challenging to assess their longer term impact quantitatively.

**TABLE 1:** Impact and effectiveness of OpenWHO IPC COVID‐19 training courses across all WHO IPC training courses.

Category	Description	Score (%)
**Course quality**	Perceived as “very high” or “high” by those who are English speaking	93–95
	Perceived as “very high” or “high” by those who are Spanish speaking	93
**Enjoyability**	Perceived as “enjoyable” by those who are English speaking	84–87
	Perceived as “enjoyable” by those who are Spanish speaking	76–77
**Usefulness**	Perceived as “very useful” by those who are English speaking	80–81
	Perceived as “very useful” by those who are Spanish speaking	67–72
**Application of learning**	English‐speaking participants felt they had used what they had learned from each of the courses	66–73
	Spanish‐speaking participants felt they had used what they had learned from each of the courses	79–82

Abbreviations: IPC, infection prevention and control; WHO, World Health Organization.

Qualitative evidence suggests that the impact of the WHO courses extended well beyond direct participants, with evidence of the training elements and materials being incorporated into other training formats. Interviews revealed that some WHO regions and national MoH staff incorporated OpenWHO courses into educational curricula or professional training, whereas others created bespoke materials to address local needs. As one participant said, “we used it [the OpenWHO courses] to enrich our own training materials that we already had.” Additionally, in the workplace, participants reported that they used their learning to develop institutional policies, to instill and monitor good practice, and to share learning with colleagues, whereas in their private lives, their learning helped to modify their own behavior and that of their families.

#### 3.1.3 RCCE Products

The numerous RCCE products were initially disseminated to the six WHO regions; in understanding their ultimate impact, it is important to explore their outcome once received at the regional level.

**FIGURE 2:**
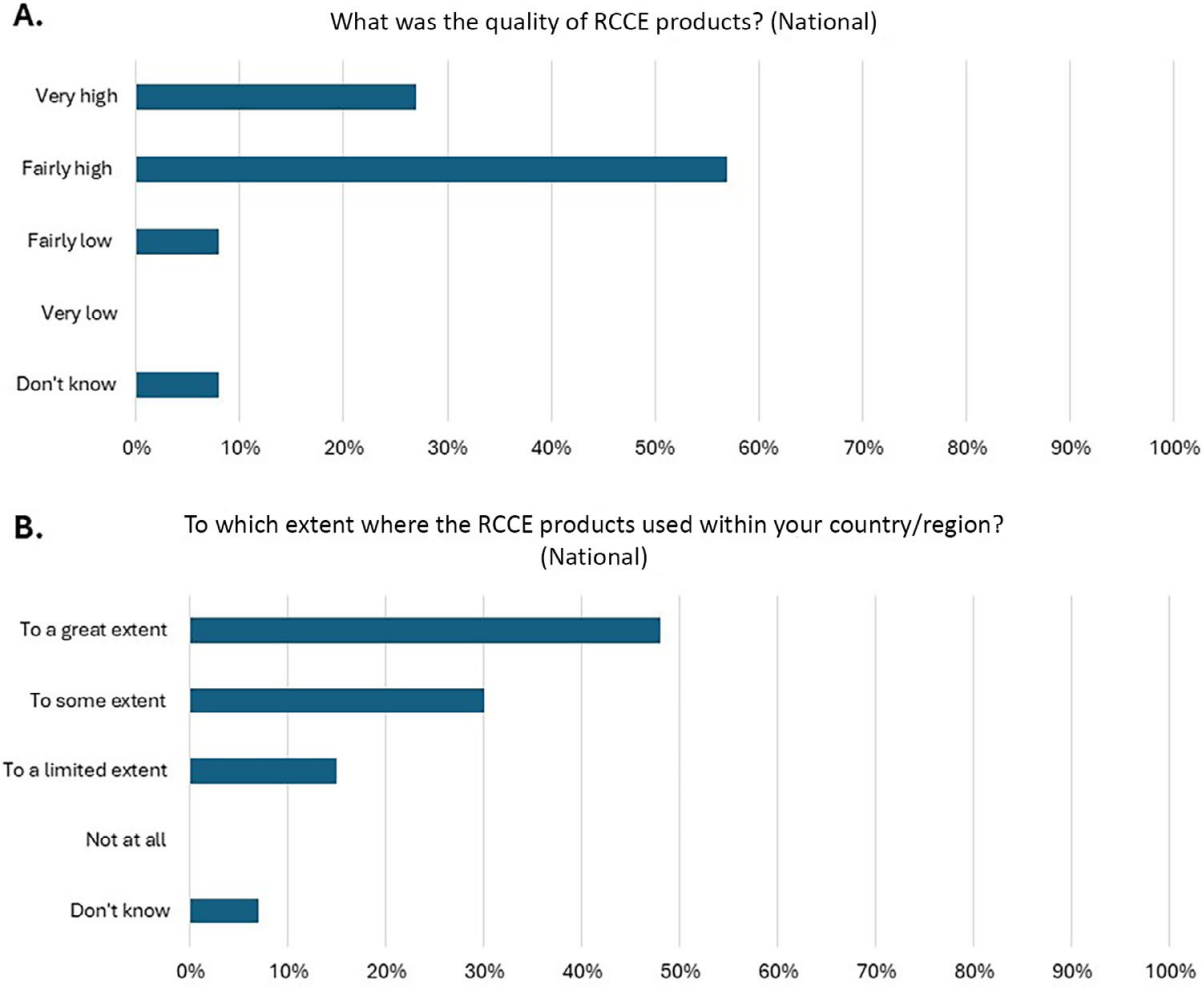
Impact and effectiveness of IPC COVID‐19 risk communications and community engagement products. In summary, WHO RCCE products were viewed as high quality, and they were widely adapted and used at the national level by survey respondents. RCCE, risk communication and community engagement. Figure 2A shows perceived quality of RCCE products. Figure 2B shows the use of RCCE products within countries/regions.

Regional IPC focal points disseminated and used the RCCE products in various ways and to differing degrees. Some regions primarily developed their own RCCE products; others adapted WHO's products, whereas a third set used these materials in their original form, as far as was possible. Given these different approaches, it is not surprising to find around half of regional and national representatives reported that WHO's RCCE products had been utilized to “a great extent,” whereas others indicated to “some” or a “limited” extent (Figure 2). Similar findings were reported on the quality of the products (Figure 2).

When describing how the RCCE products were used, national representatives referred to them being disseminated through various media, including radio, television, and social media. Individuals described how the products were used to create materials that were distributed or presented to communities in a range of settings, both physically and orally. When asked what changes, positive or negative, the RCCE products had generated for individual countries, national representatives identified raised awareness, both of RCCE as a discipline of public health among officials and of COVID‐19 symptoms and mitigation strategies among communities. Such awareness was seen to lead to changes in behavior and practice.

Although feedback and impact tracking primarily occurred during the product development and testing stages, no data were collected at global or regional levels regarding the actual impact of RCCE products. Once a product was shared, data on impact focused on its usage and to the extent that organizations at the regional and national levels perceived its effectiveness. Feedback from national representatives suggested that as part of a package, culturally contextual RCCE materials contributed to increased awareness and changes in public behaviors, specifically around hand hygiene and social distancing.

This study found substantial evidence that the products codeveloped by the WHO's COVID‐19 IPC and RCCE response teams were accessed and utilized at regional and local levels, resulting in impacts on knowledge, practice, and behavior. However, this impact was uneven, with different regions adopting the WHO's RCCE products and accessing the WHO training to varying degrees.

#### 3.1.4 The Overall Picture

Inevitably, any attempt to evaluate the impact of individual products or types of products, in the context of a global pandemic with unprecedented flows of information, is highly problematic. Nonetheless, the study unearthed a far greater focus on IPC in many member states compared to pre‐pandemic, which was attributed to the cumulative impact of the WHO's response products. Many practitioners at the national level emphasized a stronger IPC culture being embedded within their institutions because of the knowledge shared via the WHO products.

### 3.2 SECTION 2: Preparing for the Next Pandemic

Despite the substantial impacts described thus far, our study revealed a range of challenges in the development, dissemination, and evaluation of the response products developed by the team. To prepare for a potential future pandemic, it is critical to address these challenges and devise solutions to enhance the impact of guidelines, training, and RCCE products. In the remainder of this article, we examine four of these challenges.

#### 3.2.1 Timeliness

Across all three pillars of work, there was evidence of a conflict between the desire to release materials quickly and the fundamental objective to ensure that these products were based on the most robust evidence available. This is inevitably a tricky balance, and one that will have consequences for both the quality of the WHO's products and the nature of their impact.

The development of the WHO's initial IPC interim guidance was based on review of available evidence and existing WHO IPC guidelines, including those for other acute respiratory infection (ARI) pathogens.^[^ ^8^, ^9^, ^10^ ^]^ The GRADE process, an established method involving a rigorous approach to using scientific evidence to answer specific “PICO” questions (defining population, intervention, control, and outcome), was implemented later in the pandemic response.^[^ ^10^ ^]^ The use of the GRADE process for assessing evidence, although rigorous, was time consuming, inevitably leading to delays in the release and availability of the latest evidence‐based guidelines and their accompanying training and RCCE products.

To expedite IPC guideline availability during infectious disease outbreaks, standard templates for IPC foundational practices should be created to serve as interim guidance for “unknown pathogens,” alongside disease‐specific IPC guidelines to ensure resources are accessible during emergencies. Greater alignment is necessary between the dissemination of guidelines, the release of RCCE products, and relevant IPC for Member States to ensure supportive materials are available upon dissemination.

#### 3.2.2 Translating Globally Developed Products Into Local Contexts

WHO IPC COVID‐19 response products were not simply received and utilized at the regional, national, or facility levels in their original form; rather, there was often an intermediary stage of interpretation or translation. For example, some regions made adaptations to RCCE materials to make them more culturally relevant, whereas many countries or organizations adapted elements of the WHO training for their own purposes. There were some impressions that WHO guidelines highlighted IPC measures within an “ideal” context, with insufficient consideration of what was feasible within an individual country or healthcare context. On the basis of these challenges, some study participants suggested that WHO headquarters should have provided guidelines more adapted to reflect different resource contexts and scenarios, an activity ultimately undertaken by individual regions, countries, and facilities—leading to additional delays in some instances in their utilization.

To foster a more IPC‐ready future at all operational levels, several reflections are proposed. First, there is a need to tailor guidelines to national contexts to ensure successful uptake and implementation, with WHO headquarters further engaging regional and national IPC focal persons to help interpret these guidelines. Additionally, national institutions responsible for developing IPC guidance should be made aware that WHO guidelines are recommendations requiring contextualization. Simplifying technical language in guidelines by producing user‐friendly versions of each guidance will make them more accessible, especially for frontline health and care workers.

To improve accessibility and engagement with training courses, several strategies should be considered. First, partnering with local NGOs or Ministries of Health to codevelop translations to local languages in underrepresented regions, while also adapting examples, case studies, and visuals to reflect local healthcare settings and challenges on the ground. This will increase relatability and comprehension. Second, expanding these partnerships to tailor outreach campaigns accordingly and to identify the most effective channels of dissemination, including popular messaging platforms. Third, equipping local training providers with frameworks and tools to help them select the best training modality for the context and needs.

#### 3.2.3 Feedback and Evaluation Mechanisms

Individuals at global, regional, and national levels reported that structures for feedback on guidelines were limited, often requiring proactivity on the part of recipients, rather than involving a systematic approach to data collection. For instance, the global team received feedback from emails, either submitted via regional focal points or media requests; as well as via social media channels, such as X (formally Twitter). The data, which WHO appeared to collect proactively and systematically, were the number of individual guideline downloads. As noted previously, the impact of the WHO training was not monitored beyond the point of course completion, making it impossible to identify how participants ultimately used what they had learned. Participants also noted that there was no systematic tracking mechanism for RCCE products within countries, measuring indicators such as reach, behavior change, or awareness. Structures and processes for monitoring the impact of specific IPC response products were limited prior to this evaluation. Consequently, it is important to explore how this could be improved in the future, such as embedding a monitoring, evaluation, and learning system into operations from the outset of every response to assess the usage and effectiveness of guidelines, training materials, and risk communication products.

Gathering feedback at a global scale is inherently challenging, especially given the scope of the WHO response efforts. With ongoing financial constraints in global health, comprehensive evaluations such as these may be viewed as too costly or time‐intensive, given competing priorities. A simplified after‐action reporting approach, focused on both technical products and response actions, may offer a more practical way to capture lessons learned within a shortened and more efficient timeline.^[^ ^11^, ^12^ ^]^

#### 3.2.4 Maintaining Behaviors and Learning

Study participants at the national level were optimistic about sustaining the behavior changes achieved by the WHO's IPC response products. Both regional and national representatives noted that IPC currently holds significantly greater status in their respective regions or countries than prior to the COVID‐19 pandemic, which they believe could support lasting change. However, participants emphasized that maintaining these IPC gains requires resources (including supervision, refresher training, availability of PPE, and working water taps, among other infrastructure) and being consistently available.

Participants also expressed concern over perceived reductions in motivation among health and care workers adhering to IPC procedures, compared to during COVID‐19, when fear was a motivating factor. To address this, capacity strengthening should extend beyond pandemic response, embedding IPC into preservice education and continuous professional development frameworks while also providing certification or Continuing Medical Education (CME) credits. In other words, to ensure the IPC standards established and/or enhanced by the WHO's response products during the pandemic remain intact for any future pandemic, proactive action is required to secure, embed, and enhance the gains made during the pandemic. The recent publication of the Global action plan and monitoring framework on IPC 2024–2030 adopted at the 77th World Health Assembly outlines actions for Member States to take to ensure sustainable IPC programmes moving forward.^[^ ^13^ ^]^

## 4 Conclusions

In conclusion, the WHO IPC COVID‐19 team has created impact amongst Member States through its work. There is strong evidence of the effectiveness and use of the IPC COVID‐19 guidelines and courses developed and shared via OpenWHO. There is weaker evidence of the impact of RCCE products/interventions as a standalone activity. Its complementary role in the delivery of training courses is, however, strong.

Key areas of impact include shifts in national policy toward a greater recognition of IPC as a vital emergency response pillar and enhanced health and care worker IPC knowledge. A more active IPC culture is evident in many health institutions, with the establishment of new or revitalized IPC committees, institutional IPC focal points, and greater levels of institutional supervision of IPC compliance. This is thanks to the implementation of the IPC core components, yet the emphasis on IPC during the COVID‐19 pandemic undoubtedly acted as a major impetus for advancing awareness, knowledge, operationalization, and reinforcing the need to institutionalize IPC.^[^ ^14^, ^15^ ^]^

Despite evidence describing the effectiveness of the WHO IPC COVID‐19 products, further research into the impact of training courses and the implementation of IPC guidelines would provide a more robust picture of the impact of this work. Additional studies exploring the methodology for tailoring RCCE products to local contexts could also enhance their impact as standalone tools. Finally, conducting after‐action reviews (and documenting the learning from these) following the acute phase of each emergency can enhance reflection, identify strengths and weaknesses, and support ongoing monitoring and evaluation efforts.

The WHO IPC teams have a strong advocacy role to play in supporting sustainability efforts nationally. This includes advocating for Member States to ensure IPC programmes, guidelines, and training for the health workforce on IPC practices are incorporated into health system preparedness,^[^ ^16^ ^]^ readiness, and response planning for future epidemics or pandemics^[^ ^13^, ^17^, ^18^ ^]^ and advocating for rigorous maintenance of health facility infrastructure and supply chains to support IPC practices. This would address concerns raised by practitioners in this study who reported observing a gradual decline in compliance with IPC COVID‐19 guidelines and practices. In the context of pandemic preparedness, it is critical for the WHO to continue to provide IPC norms and standards, training, and communication products. Evaluations such as these should be incorporated to ensure transparency and effective use of resources, which are pertinent in the current global health financial constraints.

## Author Contributions

**Femi Nzegwu**: conceptualization, writing – original draft, methodology, writing – review and editing, formal analysis, project administration, supervision, validation, data curation. **Hannah Hamilton Hurwitz**: conceptualization, funding acquisition, writing – review and editing, project administration, validation, visualization. **Victoria Willet**: conceptualization, funding acquisition, writing – review and editing, validation, project administration. **Elizabeth Clery**: investigation, writing – review and editing, methodology, formal analysis, data curation. **Maryirene Ibeto**: formal analysis, writing – review and editing, methodology, investigation, data curation. **Joanna Wild**: investigation, writing – review and editing, methodology, formal analysis, data curation. **April Baller**: conceptualization, funding acquisition, writing – review and editing, validation, supervision, project administration.

## Ethics Statement

Ethical approval for this evaluation was waived by the London School of Hygiene and Tropical Medicine in line with its research governance. LSHTM's policy requires only research (compared to this service evaluation) to be submitted to the Research Ethics Committee for review.

## Conflicts of Interest

We disclose that members of the World Health Organization (WHO) Infection Prevention and Control, Water, Sanitation, and Hygiene (IPC/WASH) team (H.H.H., V.W., and A.B.) were interviewed for this study due to their roles in the COVID‐19 pandemic response. To maintain objectivity, these individuals did not participate in the analysis of the interviews. Three authors (H.H.H., V.W., and A.B.) are employed by the WHO. The remaining authors (F.N., E.C., M.I., and J.W.) received funding from the WHO to conduct this research.

## Data Availability

Given the predominantly qualitative nature of this study's data, most with stakeholders where only one individual holds a position, either within federal or state government, facilities, NGOs, or global or country‐based international agencies; and the small sample size of the survey primarily from a select number of countries and organizations, the transcript and survey data analyzed in this study will not be publicly accessible because this would violate the conditions of informed consent. Every care has been taken to ensure anonymity of the data in the submitted manuscript. For enquiries about data access, please email researchdatamanagement@lshtm.ac.uk.
